# Publication of registration studies for veterinary medicine in peer-reviewed academic journals

**DOI:** 10.18849/ve.v9i3.682

**Published:** 2024-09-18

**Authors:** Jonathan Elliott

**Affiliations:** RCVS Knowledge London, UK

**Keywords:** drug registration, pharmaceutical companies, academic publishing

## Abstract

No matter where you are in the world, you have unrestricted access to the evidence published in Veterinary Evidence. You can submit to, read and share our peer-reviewed content for free and use it to enhance the quality of care you provide to animals.

## Introduction

Much clinically applied research undertaken by research and development departments of pharmaceutical companies is used to support the registration of veterinary medicinal products but is only made publicly available in summary form by the authorisation bodies (e.g. European Medicines Agency or U.S. Food and Drug Administration Center for Veterinary Medicine). Many veterinary practitioners do not read these summaries and are not fully aware of what is required to gain a product authorisation. When pivotal registration trials are published in peer-reviewed academic journals some might think this is all the research that underpins the authorisation of the product.

This could not be further than the truth. In order to gain a marketing authorisation for a veterinary medicinal product, the company needs to have demonstrated that the product:

- can be manufactured to highly reproducible standards (quality of product);
- has a favourable risk-benefit analysis which supports its proposed clinical use such that if used as per the datasheet indications, the patients treated (and owners administering the treatment) should not be harmed by its administration. Any patient groups at higher risk of suffering adverse effects will be identified by the datasheet warnings and so excluded from receiving treatment. Furthermore, they need to show the environment is not harmed by the use of the product (safety of the product);
- is efficacious and the evidence supporting the indications claimed on the datasheet is robust (efficacy of the product).

## Registration Dossier Requirements

Registration dossiers containing the evidence for the quality, safety and efficacy of new veterinary medicinal products are substantial, usually contain multiple studies undertaken over several years, and each part is evaluated by trained assessors who can have access to all the raw data from all the studies companies have conducted. All pivotal studies are expected to have been undertaken to a quality standard that is agreed internationally to be to Good Manufacturing Practice (GMP), Good Laboratory Practice (GLP), or Good Clinical Practice (GCP) standards. Published VICH [1] guidelines explain what is expected in each of these international standards. Principles of GCP and GLP relevant to all pivotal preclinical and clinical studies under-pinning the safety and efficacy of a product include producing an agreed study protocol ahead of commencing the study. The protocol should be clear and strictly adhered to with any protocol violations or amendments that occur during the study clearly documented and explained in the final study report. This protocol will define and justify the study’s primary efficacy end-points, explaining their relevance to the general patient population, how they will be measured, and how the test and control treatments will be compared. It is standard practice to also provide a plan for statistical analysis alongside the study protocol which justifies the number of patients to be included in each group and identifies the statistical methods to be used.

Because assessors can have access to the raw data, they can check that the inclusion and exclusion criteria were adhered to, and the protocol has been correctly followed for each patient. Indeed, quality audits undertaken by the companies should have identified any anomalies prior to submission and corrected these if they are found. In addition, measurement methods (e.g. drug plasma concentration assays, hormone evaluation assays) used in these studies are expected to be fully validated and evidence of validation and the quality assurance assessments carried out and will need to be presented as part of the data package submitted within the dossier.

Furthermore, for EU submissions, companies are required to include detailed and critical assessments of the studies submitted (‘expert report’) which are written by suitably qualified individuals who are independent of the study investigator and are often not an employee of the company, albeit they are engaged by the company on a consultancy basis. Expert reports are required for each part of the dossier. This provides the company with an independent review of their data ahead of submission of the dossier for registration purposes and identifies where any scientific weaknesses lie. This critical evaluation may lead to further studies being undertaken or datasheet claims, contraindications, or warnings being changed.

## Publication in Academic Journals

When registration studies are presented to peer-reviewed academic journals for publication, the data included needs to conform to the journal’s requirements in terms of word length of the manuscript (often 4000 to 5000 words). In many cases this limits the amount of data and level of description that can be included in the manuscript such that many details have to be omitted.

Thus, any published manuscript will be much less detailed than the dossier required for registration purposes and will generally not include the ancillary (non-pivotal) studies that informed the design of the pivotal field safety and efficacy (FSE) study or the quality assurance data that underpinned the measurement methodology used. These ancillary studies would normally include research that determines and justifies the dose to be used in the pivotal FSE study. This may involve an experimental model of the disease to be treated in the field combined with pharmacokinetic (absorption, distribution, metabolism and elimination (ADME) studies), pharmacodynamic, and toxicology studies, and preliminary margin of safety studies in the target species.

Some journals do allow submission of supplementary data which does not count in terms of the final word count of the manuscript, but this would not usually extend to experiments that might constitute another full manuscript as would be the case for dose determination studies. Most do not require submission of the raw data from individual animals that were included in the study or the quality assurance data to be made available to the reviewers. Reviewers of academic papers are not paid for the time they spend undertaking the review process nor do they have access to the raw data to undertake a complete evaluation of the study, hence the review process relies on accurate and faithful recording, inclusion, and interpretation of data by the authors of the paper. Peer review relies on the time and diligence of the reviewer who can only assess the data and the inferences made by the authors of the paper by benchmarking them against previous publications and the reviewers’ subject knowledge. This is very different from the processes undertaken by regulatory authorities with access to all the data, which undergoes a much greater degree of scrutiny and validation.

## Target Animal Safety Studies

Safety of any registered veterinary medicinal product is of paramount importance, yet it is difficult to prove a medicine is without adverse effects before it is authorised. This is why the assessment of risk-benefit is made, taking account of all the data available for the particular product at the time. The pivotal target animal species (TAS) safety study is just one piece of evidence presented in the registration dossier and sometimes these studies will also be submitted to peer-reviewed academic journals for publication. Usually, these studies are neither exciting nor innovative science, but nevertheless they are an important part of the risk-benefit analysis. Unlike the pivotal FSE study, these studies are undertaken in fit healthy, usually young experimental animals of the target species. Their design depends on the dose and duration of treatment that is to be used in the FSE study. Usually these studies examine 1x, 3x and 5x the recommended therapeutic dose administered for at least the maximum recommended duration of treatment or, if the treatment is intended to be chronic, for up to 6 months. A placebo group is included to give the background level of spontaneous adverse events or pathological lesions found in animals of this age.

It is important to recognise that the TAS safety study is not powered to demonstrate statistical significant effects against a placebo. The minimum number of animals is used to reduce the sacrifice of healthy experimental dogs or cats for this purpose in alignment with the principles of the 3Rs (Replacement, Reduction, Refinement). International guidelines on the design and conduct of TAS safety studies have been published. Group sizes are typically four males and four females per dose group and the pattern of effects on particular organ structure or function will be assessed over time by undertaking comprehensive haematology and serum biochemistry testing at regular intervals over the dosing period and assessing organ pathology at the end of the study (both gross and histopathology). If product-related effects are evident, they are usually dose and / or exposure time-related. Idiosyncratic reactions are very unlikely to be identified in registration studies. Toxicokinetic studies are increasingly being used to identify drugs which accumulate within the body and will be complemented by the basic pharmacokinetic studies undertaken in the target species. Such dose and time-dependent signals from the TAS will be taken seriously, particularly if they are seen in the majority of the animals tested and are deemed likely to be clinically significant (regardless of whether statistically significant changes are seen). These signals will inform the monitoring, interpretation and analysis of safety data (suspected adverse drug effects) from the FSE study and feed into the risk-benefit analysis.

Many of the veterinary medicinal products that are being developed will have been widely screened in pre-clinical development to understand selectivity of receptor binding, what metabolites are formed, whether they have mutagenic potential, and so on. They will also have been tested for toxicity in laboratory animals and, when the same class of active ingredient is in use in humans, information on adverse effects seen will also be used in designing and interpreting the TAS study. If, for example, the active ingredient has effects on the electrocardiogram (ECG) in people, ECG analysis will be part of the TAS study or a specific study assessing cardiac effects will be conducted. Thus, if TAS studies are submitted to peer-reviewed academic journals for publication, it should be recognised that they are just part of the information which is used to inform the risk-benefit analysis of a specific veterinary product.

## Pharmacovigilance

Assessment of safety (and efficacy) of a particular Veterinary Medicinal Product does not end with the product authorisation based on the pivotal TAS and FSE studies. Pharmacovigilance is an important aspect of the assessment of the safety of authorised products. This is dependent on veterinary professionals reporting suspected adverse events (including lack of efficacy) when they encounter them in individual animals they treat with the medicinal product. These are reportable to the market authorisation holder and to the body issuing the product authorisation (in the UK that would be the Veterinary Medicines Directorate). Providing as much information as possible about the case will facilitate the pharmacovigilance team’s ability to recognise important patterns in the data they receive from the field. Companies are required to share any reports they receive, and Medicines Agencies monitor these data continuously and may require investigation as and when patterns of adverse effects or reports of lack of efficacy emerge.

## Conclusion

In conclusion, it is desirable for companies to publish pivotal clinical trial data on new products in peer-reviewed academic journals so that veterinary practitioners can read part of the evidence underpinning the claims of efficacy and that is used in the risk-benefit analysis that was undertaken prior to granting a product authorisation. When reading these publications, practitioners should recognise that the FES and TAS studies, whilst pivotal to the authorisation, are supported by many other studies that underpin their design and interpretation and provide assurance of the quality of the data. Most of these supporting studies will not be presented alongside the pivotal studies in peer-reviewed academic journals but were very much an important part of the registration dossier submitted when applying for a marketing authorisation.
